# Parasites and genetic diversity in an invasive bumblebee

**DOI:** 10.1111/1365-2656.12235

**Published:** 2014-06-03

**Authors:** Catherine M Jones, Mark J F Brown, Thomas Ings

**Affiliations:** School of Biological Sciences, Royal Holloway, University of LondonEgham, TW20 0EX, UK

**Keywords:** *Apicystis*, biological invasion, *Bombus hypnorum*, enemy release, parasite acquisition, *Sphaerularia*

## Abstract

1. Biological invasions are facilitated by the global transportation of species and climate change. Given that invasions may cause ecological and economic damage and pose a major threat to biodiversity, understanding the mechanisms behind invasion success is essential.
2. Both the release of non-native populations from natural enemies, such as parasites, and the genetic diversity of these populations may play key roles in their invasion success.
3. We investigated the roles of parasite communities, through enemy release and parasite acquisition, and genetic diversity in the invasion success of the non-native bumblebee, *Bombus hypnorum*, in the United Kingdom.
4. The invasive *B. hypnorum* had higher parasite prevalence than most, or all native congeners for two high-impact parasites, probably due to higher susceptibility and parasite acquisition. Consequently parasites had a higher impact on *B. hypnorum* queens’ survival and colony-founding success than on native species. *Bombus hypnorum* also had lower functional genetic diversity at the sex-determining locus than native species. Higher parasite prevalence and lower genetic diversity have not prevented the rapid invasion of the United Kingdom by *B. hypnorum*. These data may inform our understanding of similar invasions by commercial bumblebees around the world.
5. This study suggests that concerns about parasite impacts on the small founding populations common to re-introduction and translocation programs may be less important than currently believed.

## Introduction

Biological invasions occur when non-native species successfully establish in a new location and rapidly expand their range (Williamson 1996). Such invasions may affect the diversity and abundance of native species, species interactions (e.g. symbioses) and the provision of ecosystem services (such as pollination), which are important for human well-being (Pimentel, Zuniga & Morrison 2005; Pejchar & Mooney 2009; Vila *et al*. 2010). The invasion success of a non-native species may be facilitated by a release from natural enemies, such as herbivores, predators and parasites, potentially leading to a rapid increase in distribution and abundance of the invasive species (Elton 1958; Keane & Crawley 2002; Torchin *et al*. 2003). Evidence supporting the enemy release hypothesis can be found from studies of plant–herbivore interactions (e.g. Agrawal & Kotanen 2003; Colautti *et al*. 2004; Agrawal *et al*. 2005; Liu & Stiling 2006) but the evidence from animal–parasite systems is less clear (e.g. Dunn & Dick 1998; MacNeil *et al*. 2003; Georgiev *et al*. 2007). Given the historical, current and predicted global impact of invasive species (Elton 1958; Vitousek *et al*. 1996; Wilcove *et al*. 1998; Pimentel, Zuniga & Morrison 2005) and the importance of species range expansion due to climate change (Parmesan *et al*. 1999; Hickling *et al*. 2006) understanding the mechanisms that facilitate these events is a key challenge (e.g. Phillips *et al*. 2010; White & Perkins 2012).

Previous studies of the role of parasites in enemy release, in both plant and animal systems, largely examine either parasite prevalence or the impact of individual parasite species (e.g. MacNeil *et al*. 2003). However, parasites exist in communities (Cloutman 1975; Holmes & Price 1986) and invasive species may host multiple parasite species (e.g. Georgiev *et al*. 2007). Interactions among parasite species, within a host, include competition for resources (Rigaud, Perrot-Minnot & Brown 2010) and alteration of transmission rates (e.g. castrating parasites reducing the transmission of other parasite species to the offspring of the host, Ben-ami, Rigaud & Ebert 2011). The presence of multiple parasite species can induce a range of host immune responses, which may have divergent impacts on individual parasite species (Schmid-Hempel 1998). In addition, the structure of parasite communities can have significant consequences for assessing the impact of individual parasites (e.g. Rutrecht & Brown 2008). Consequently, understanding the structure of parasite communities and their subsequent impact (Rigaud, Perrot-Minnot & Brown 2010) is essential to establish the role of parasites in invasions.

While invading species may be released from parasites in their new location, the impact of parasites in the invaded communities may, in turn, be modified by invasive species. This may occur through parasite introduction (Dunn 2009) or parasite spillover (Daszak, Cunningham & Hyatt 2000; Kelly *et al*. 2009) where invading host species introduce non-native parasites and these spillover to infect native hosts. Invasive species may also acquire parasites from congeneric host species in the new location (parasite acquisition: Dunn 2009) which may result in an increase (through invasive species acting as a reservoir for native parasites followed by parasite spillback; reservoir host: Norman *et al*. 1999; Daszak, Cunningham & Hyatt 2000; Dunn 2009; Kelly *et al*. 2009; parasite spillback: Daszak, Cunningham & Hyatt 2000; Kelly *et al*. 2009) or a decrease in parasite abundance in native species (through parasite dilution, where invading hosts provide an additional or alternative host for native parasites ‘diluting’ the parasite prevalence and/or abundance in native hosts: Norman *et al*. 1999; Ostfeld & Keesing 2000) depending on the competence of the invasive host at transmitting the infective stages of the parasite. These factors may occur individually or in concert, and thus investigating enemy release in the invaded range should take account of these complex interactions.

An additional factor that may play a key role in the host–parasite interactions of invasive species is the genetic diversity and provenance of the invasive host. Invasive species are likely to establish in a new location from only a few propagules or reproductive individuals, and therefore the founding population will have low genetic diversity (Dlugosch & Parker 2008). Low genetic diversity in natural populations is known to be associated with higher rates of parasitism (e.g. Whitehorn *et al*. 2011) and thus genetically depauperate invasive species may be more likely to acquire parasites from congeners. In addition, invading hosts have not co-evolved with native parasites. Consequently, invading hosts may be maladapted to native parasites and these parasites may therefore have a greater (or lesser) impact on such hosts (Thompson 2005). Relative to native hosts, if the non-native species is less susceptible to parasites and/or these parasites have a smaller impact on fitness, non-native hosts are likely to benefit from enemy release despite the acquisition of generalist parasites from congeners.

While bumblebees (*Bombus* spp.) are generally considered to be in decline (Goulson, Lye & Darvill 2008; Williams & Osborne 2009), which is concerning as they are important ecological and commercial pollinators, they can also be highly invasive (Dafni 1998; Goulson 2003). In Japan, commercially introduced *Bombus terrestris* L. have escaped and threaten native congeners and their interactions with native plants (Matsumura, Yokoyama & Washitani 2004; Inoue, Yokoyama & Washitani 2008). Invasive *B. terrestris* have spread throughout Tasmania in the last 20 years (Allen *et al*. 2007; Schmid-Hempel *et al*. 2007) probably from New Zealand, where they were introduced in the 19th century (MacFarlane & Griffin 1990). Most recently, invasive *B. terrestris* has spread across Argentina and Chile, where it is blamed for rapid declines in the only native bumblebee species, *Bombus dahlbomii* Guérin-Méneville (Torretta, Medan & Abrahamovich 2006; Plischuk & Lange 2009; Goulson 2010; Arbetman *et al*. 2013; Morales *et al*. 2013).

Using the successful establishment of a non-native invasive bumblebee, *Bombus hypnorum* L., across England and Wales over the last decade (Goulson & Williams 2001; BWARS), we aim to identify the role of parasites and genetic diversity in this invasion. *Bombus hypnorum*, the tree bumblebee, has expanded across England, Wales and Scotland, to Lennoxtown, Scotland (*c*. 600 km), to Truro, Cornwall in the South West (*c*. 300 km) and Pembrokeshire in Wales (*c*. 320 km) since its first discovery in the New Forest, Wiltshire, England in 2001 (BWARS, Goulson & Williams 2001). The parasite community of bumblebees is composed of generalist parasites and has been well characterized (MacFarlane, Lipa & Liu 1995; Schmid-Hempel 1998; Rutrecht & Brown 2008), making this an excellent opportunity to examine how enemy release and parasite acquisition may impact an invasive species, particularly as recent work has suggested that nest parasites play a role in the dynamics of native bumblebee populations (Antonovics & Edwards 2011).

Enemy release can occur in two ways. First, an invading species, in the invaded range, may escape from the enemies it would have encountered in its native range. A model proposed by Drake (2003) suggests that such enemy release may be important for the establishment of small invading populations. Second, invading species may escape from enemies present in the invaded range, as those enemies are not adapted to exploit it (Dunn 2009). A comparison of enemies of invading species and those of congeneric native species investigates the second mechanism and we take this approach because the origin of our focal species is currently unknown. To investigate the potential release from natural enemies of the non-native *B. hypnorum*, we determined the parasite community in queens of this invasive bumblebee species and compared it to those of five native bumblebee species with the expectation that *B. hypnorum* would have lower parasite prevalence and lower parasite species richness than native congeneric species, and thus it should be released from its parasite enemies. In addition to investigating parasite prevalence, parasite species richness and parasite community structure, we also investigated the parasite impact on a proxy for host fitness, and functional genetic diversity at the sex-determining locus, in laboratory-reared colonies of *B. hypnorum*. We expected that parasites would have a greater impact on fitness in *B. hypnorum* than in native congeneric species and that the genetic diversity of *B. hypnorum* would be lower than that of native *Bombus* species.

## Methods

### Biology of the study system

Most bumblebees are annual eusocial species, passing through a solitary overwintering phase as queens. This makes the queen a key component of the annual life cycle. Interestingly, bumblebee queens are particularly heavily impacted by parasites (Rutrecht & Brown 2008). Consequently, parasites that reduce the survival and colony-founding success of the queen are likely to have a high impact on bumblebee populations and, therefore, in this study we focused on bumblebee queens. The ultimate measure of parasite impact on fitness would be the proportion of sexuals produced by colonies that contribute their genes to the subsequent generation. However, such an analysis is logistically extremely challenging and beyond the scope of this study. Bumblebee gynes (unmated new queens) disperse from their natal nests to mate in late summer, prior to finding a hibernation site. Queens hibernate in individual hibernacula, which can be dispersed or aggregated, depending on the species, and different species favour different hibernation sites (Alford 1969b; Sladen 1912). Variation in hibernation sites may impact the probability of infection by some parasite species (see below) but too little is known about hibernation site choice to make any predictions. Post-hibernation queens disperse again, with estimates of aggregate dispersal of at least 5 km (Lepais *et al*. 2010), and congregate at florally rich sites to forage for nectar and pollen. Parasites can be acquired from natal nests, interactions with males during mating, during hibernation and through foraging pre- and post-hibernation (Schmid-Hempel 1998).

### Sampling scheme

Our sampling methodology was designed around the biology of the system (see above). Bumblebee queens were collected, between February and May 2011, from two primary florally rich sites in Surrey and Berkshire, Windsor Great Park (Lat. 51·41, Long. −0·60) and the Royal Horticultural Society (RHS) Garden, Wisley (Lat. 51·32, Long. −0·58). Additional queens were collected from florally rich sites at the Royal Botanic Gardens, Kew (Lat. 51·47, Long. −0·30); Royal Holloway, University of London (RHUL) (Lat. 51·43, Long. −0·56) and Horsell, Surrey (Lat. 51·32, Long. −0·57). Our sampling area was geographically restricted due to the requirement to catch sufficient queens within a limited time period. However, due to the rapid establishment of this invasive species in the United Kingdom, we believe that the population in South East England is likely to be representative of the UK *B. hypnorum* population as a whole. The non-native species *B. hypnorum* and five native species *B. jonellus* Kirby*, B. pratorum* L.*, B. lucorum* L.*, B. pascuorum* Scopoli and *B. terrestris* were collected. The queens were collected using an entomological net and placed in individual plastic vials in a chilled container and transported to RHUL. On each day, sites were collected to exhaustion. The queens were spring queens, foraging after emerging from hibernation, and therefore from the first voltine generation. While abundant species may be the most obvious source of generalist parasites, such parasites are also more likely to infect related host species (Perlman & Jaenike 2003), and our sampling strategy was designed to cover both possibilities, with *B. jonellus* and *B. pratorum* being the phylogenetically closest relatives to the invasive *B. hypnorum* (Cameron, Hines & Williams 2007) and *B. lucorum, B. pascuorum* and *B. terrestris* being the most abundant native bumblebee species (Goulson & Darvill 2004; Goulson *et al*. 2005; Williams 2005).

### Parasite – faecal check

Faecal samples were taken and examined using a ×400 phase contrast microscope for the following parasites: *Sphaerularia bombi* Dufour*,* a nematode worm; *Apicystis bombi,* a neogregarine; *Crithidia bombi,* a trypanosome; and *Nosema bombi* Fantham & Porter*,* a microsporidian. All these parasite species can be reliably identified as patent infections using microscopic techniques (e.g. Otterstatter & Thomson 2006; Rutrecht & Brown 2008). While multiple *Crithidia* spp. have been identified, molecular data show that only *Crithidia bombi* occurs in this area (M.J.F. Brown unpublished data). These are all generalist parasites with a global distribution (MacFarlane, Lipa & Liu 1995; Schmid-Hempel 1998) and both *S. bombi* and *A. bombi* have previously been reported in *B. hypnorum* (MacFarlane, Lipa & Liu 1995). Hereafter, we refer to these using their generic names. *Sphaerularia* infects bumblebee queens hibernating in the soil, castrating them and preventing them from founding colonies (Alford 1969a,b; Poinar & van der Laan 1972) and *Apicystis* kills bumblebee queens before they are able to found colonies (Rutrecht & Brown 2008). Consequently, both of these parasites have a high impact on spring queens. *Crithidia* reduces overall colony fitness by, on average, 40% (Brown, Schmid-Hempel & Schmid-Hempel 2003a), and *Nosema* has similar effects (Otti & Schmid-Hempel 2007; Rutrecht & Brown 2009).

### Parasite – dissection

The *B. jonellus, B. pratorum* and *B. pascuorum* queens were sacrificed by freezing after the faecal check and stored at −80 °C. They were later thawed, dissected and checked again for bumblebee parasites including *Sphaerularia*,*Apicystis*,*Crithidia, Nosema* and *Locustacarus buchneri* Stammer. *L. buchneri* is a tracheal mite whose impact on queens is currently unknown, although correlative studies on males and workers show lethargy and the cessation of foraging in workers of *B. bimaculatus* and reduced life span in *B. occidentalis* (Husband & Sinha, 1970; Otterstatter & Whidden 2004).

### Bee husbandry

*Bombus hypnorum* queens were reared in the laboratory at a controlled temperature (25–27 °C) and humidity (50–60%), and received sugar-water and pollen *ad libitum*. The queens were kept in the dark and a red light was used for working. Queens were kept in queen-rearing boxes, with a sugar-water dispenser and a pollen ball to encourage egg-laying. Records were kept of the reproductive output *of B. hypnorum* queens including eggs laid, number of workers, males and gynes (new queens) produced. Dead queens, either at natural death or at sacrifice, were stored at −80 °C. Queens with no offspring were sacrificed and frozen after 10 weeks in the laboratory. The queens were thawed, dissected and checked for parasites as above.

Sterile procedures were used when handling queens in the laboratory, to prevent cross-contamination. Nevertheless, two *B. hypnorum* queens that were infected by *Crithidia bombi* when dissected, but were not infected when the faeces samples were examined, were consequently rejected from the data set due to possible cross-contamination.

Queens of *B. terrestris* and *B. lucorum* were reared for other experiments by another researcher but we were still able to assess their parasite status (as described above) and whether they produced normal or diploid male colonies (see below).

### Diploid males

Bumblebees are haplodiploid, females being diploid (heterozygous) and males haploid (hemizygous). However, diploid (homozygous) males occur in inbred or genetically depauperate populations, and are indicative of low genetic diversity (Duchateau, Hoshiba & Velthuis 1994). A standard protocol for identifying diploid male production is through the presence of males in the first brood (which is usually just females) at a 50:50 sex ratio (Gerloff & Schmid-Hempel 2005). Consequently, the timing of male production was recorded to assess whether colonies were producing diploid males (Duchateau, Hoshiba & Velthuis 1994).

### Analyses

Parasite prevalence was calculated by dividing the number of infected queens by the total number of queens of each species, with 95% confidence intervals using the Clopper–Pearson ‘exact’ method. Here, we report only parasite prevalence, as for the macroparasite *Sphaerularia* the impact of an individual worm is the same as the impact of multiple worms (Alford 1969a), and nothing is known about whether variation in microparasite infection intensity affects host fitness. The parasite prevalence data and parasite impact on colony-founding data were analysed using Binary Logistic Regressions with the parasite (or parasite impact) as the dependent variable, bumblebee species and site as categorical variables with *B. hypnorum* set as the indicator species, and the forward log ratio procedure. All analyses were conducted twice, once with the entire data set and once with just the two main sampling sites (Windsor and Wisley), as these were where most of the queens were collected.

Parasite species richness, the number of parasites species in each of the parasite communities (where each bee species is a habitat that hosts a parasite community and each individual bee is a site within that habitat), and the similarity of those parasite communities were analysed using spade (Species Prediction And Diversity Estimation) software (Chao & Shen 2010).

As a measure of genetic diversity at a functionally important locus, the sex-determining locus, we estimated the number of sex alleles in the native and invasive bumblebee populations, and in a continental European population of *B. hypnorum* (data from Brown, Schmid-Hempel & Schmid-Hempel 2003b), using the formula θ = 2/*N* where ‘θ’ is the probability of a diploid colony and ‘*N*’ is the number of sex alleles (Adams *et al*. 1977; Duchateau, Hoshiba & Velthuis 1994) (and differences tested using Fisher Exact tests). The minimum number of sex alleles was estimated by comparing the number of observed and expected diploid male colonies for a range of values, and determining where they cease to be significantly different.

Statistical analyses of data were performed using IBM spss 19 for Windows and spade (Species Prediction And Diversity Estimation) software (Chao & Shen 2010).

## Results

A total of 378 bumblebee queens, collected in 225 h across 45 days, were examined for parasites (59 *B. hypnorum*, 47 *B. jonellus*, 104 *B. pratorum,* 50 *B. pascuorum,* 61 *B. lucorum* and 57 *B. terrestris*) and five parasite species were found (*Sphaerularia*,*Apicystis*,*Crithidia, Nosema* and *Locustacarus*).

### Parasite prevalence

**Figure 1:**
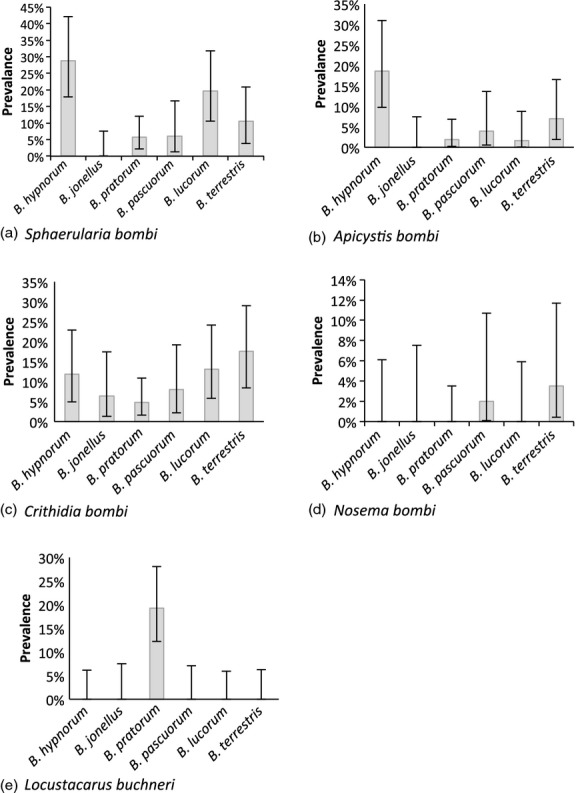
The percentage prevalence of the five parasite species across the native and non-native host species, calculated using the number of infected queens divided by the total number of queens for each *Bombus* species with 95% confidence intervals: (a) *Sphaerularia bombi*, (b) *Apicystis bombi,* (c) *Crithidia bombi,* (d) *Nosema bombi* and (e) *Locustacarus buchneri*.

The prevalence of *Sphaerularia* among bumblebee species differed significantly (Wald = 25·584, d.f. = 5, *P* < 0·001) and ranged from 29% in *B. hypnorum* to 0% in *B. jonellus* (Fig. 1). The prevalence of *Sphaerularia* in *B. hypnorum* was significantly higher than its prevalence in *B. jonellus* (Wald = 7·281, d.f. = 1, *P* = 0·007, ExpB = 0·058), *B. pratorum* (Wald = 12·623, d.f. = 1, *P* < 0·001, ExpB = 0·156), *B. pascuorum* (Wald = 10·051, d.f. = 1, *P* = 0·002, ExpB = 0·089) and *B. terrestris* (Wald = 7·416, d.f. = 1, *P* = 0·006, ExpB = 0·205) but not significantly higher than in *B. lucorum* (20%, 12/61; Wald = 0·921, d.f. = 1, *P* = 0·337, ExpB = 0·654). The prevalence of *Sphaerularia* across sites differed significantly overall (Wald = 11·887, d.f. = 4, *P* = 0·018) but in pairwise comparisons, the only significant difference was between Windsor and Horsell (Wald = 8·633, d.f. = 1, *P* = 0·003). The remaining sites, Wisley (Wald = 3·147, d.f. = 1, *P* = 0·076), Kew (Wald = 0·697, d.f. = 1, *P* = 0·404), and RHUL (Wald = 1·457, d.f. = 1, *P* = 0·227), did not differ significantly to our primary site (Windsor). The prevalence of *Sphaerularia* was not affected by collection date (this variable was not present in the final model). Qualitatively similar results were found when analyses were restricted to data from the two main sites (Windsor and Wisley; data not shown).

As with *Sphaerularia*, the prevalence of *Apicystis* among bumblebee species differed significantly (Wald = 18·927, d.f. = 5, *P* = 0·002) and ranged from 18% in *B. hypnorum* to 0% in *B. jonellus* (Fig. 1). The prevalence of *Apicystis* in the non-native *B. hypnorum* was significantly higher than its prevalence in *B. jonellus* (Wald = 4·841, d.f. = 1, *P* = 0·028, ExpB = 0·095), *B. pratorum* (Wald = 9·216, d.f. = 1, *P* = 0·002, ExpB = 0·090), *B. pascuorum* (Wald = 6·120, d.f. = 1, *P* = 0·013, ExpB = 0·108), *B. lucorum* (Wald = 6·080, d.f. = 1, *P* = 0·014, ExpB = 0·072) and *B. terrestris* (Wald = 4·416, d.f. = 1, *P* = 0·036, ExpB = 0·244). The prevalence of *Apicystis* across sites did not differ significantly overall (Wald = 6·454, d.f. = 4, *P* = 0·168) and was not affected by the collection date. Again, results were qualitatively similar in the site-restricted analysis.

*Crithidia* was the only parasite found in all six bumblebee species and prevalence ranged from 18% in *B. terrestris* to 5% in *B. pratorum* (Fig. 1). The prevalence of *Crithidia* among bumblebee species did not differ significantly (Wald = 6·846, d.f. = 5, *P* = 0·232). The prevalence of *Crithidia* across sites did not differ significantly overall (Wald = 7·722, d.f. = 4, *P* = 0·102) and, once again, was not affected by the collection date. Again, these results were qualitatively similar in the analysis restricted to the main sampling sites.

*Locustacarus* was only present in one of the six bumblebee species sampled, *B. pratorum*, with a prevalence of 16% (*N* = 104), and *Nosema* was only present in two *B. terrestris*, with a prevalence of 4% (*N* = 57) and one *B. pascuorum* queen, with a prevalence of 2% (*N* = 50).

### Parasite species richness

**Figure 2:**
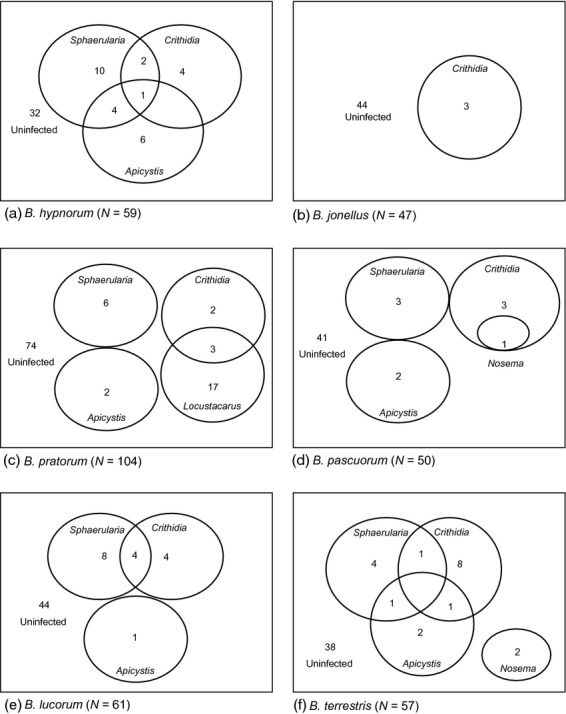
Diagrams of parasite community structure in queens of the six *Bombus* species showing overlaps where multiple infections occur: (a) *B. hypnorum*, (b) *B. jonellus*, (c) *B. pratorum*, (d) *B. pascuorum,* (e) *B. lucorum* and (f) *B. terrestris*. (Size of ovals is not representative of numbers).

**Figure 3:**
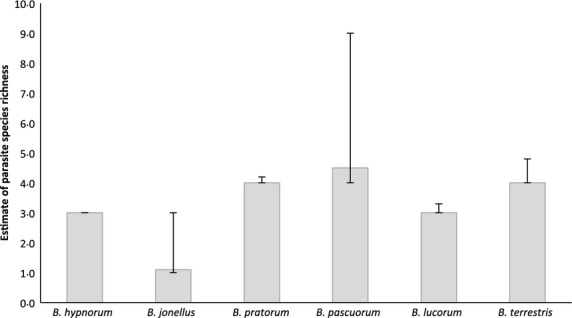
Estimate of parasite species richness (showing 95% confidence intervals) for non-native and native *Bombus* queens, calculated using spade (Chao & Shen 2010).

Observed parasite species richness differed among the sampled bumblebee species (Kruskal–Wallis *H* = 24·764, d.f. = 5, *P* < 0·001, *N* = 378) and ranged from zero to three parasite species (Fig. 2). Using the spade estimated species richness the number of parasite species in the non-native *B. hypnorum* (3·0 species, 95% CI = 3·0–3·0) was between the estimate for *B. jonellus* (1·1 species, 95% CI = 1·0–3·0) and *B. pascuorum* (4·5 species, 95% CI = 4·0–9·0) (Fig. 3).

### Parasite community structure

In contrast to our expectations that the invasive *B. hypnorum* may have escaped from its parasite enemies, the parasite communities across the non-native and native *Bombus* species were similar overall (‘Morista similarity’ multiple community measure = 0·597). Interestingly, in pairwise comparisons between the invasive species and the native species, *B. hypnorum* was more similar to the common species *B. pascuorum* (0·998), *B. lucorum* (0·917) and *B. terrestris* (0·898) than to the closely related species *B. jonellus* (0·295) or *B. pratorum* (0·360).

### Parasite impact on longevity and colony-founding

As expected, *Apicystis* in *B. hypnorum, B, terrestris* and *B. lucorum* was associated with shorter longevity post-capture (*U* = 464·000, *P* < 0·001, *N* = 177). As found in other studies, queens infected with *Apicystis* did not found a colony or produce any offspring (Rutrecht & Brown 2008). The mean post-capture life span of *Bombus* queens infected with *Apicystis* was 12·31 days (±7·786 SD, *N* = 16) and for uninfected queens 52·91 days (±35·55 SD, *N* = 161). *Sphaerularia* completely inhibited colony foundation in the two native species, as expected. However, the impact of *Sphaerularia* on *B. hypnorum* differed: five of the *B. hypnorum* queens (29%, *N* = 17) infected with *Sphaerularia* laid eggs (two produced live offspring) and this differed significantly from the expectation that no queens infected with *Sphaerularia* would lay eggs (χ^2^ = 5·8621, d.f. = 1, *P* = 0·0155). Due to sample sizes, we were not able to assess differences in the impact of the remaining, less abundant parasites. However, previous studies suggest that these have little effect on field caught spring queens (e.g. *Crithidia,* Shykoff & Schmid-Hempel 1991). Consequently, from hereon we focus on these two high-impact parasites, *Apicystis* and *Sphaerularia*.

### Parasite community impact

**Figure 4:**
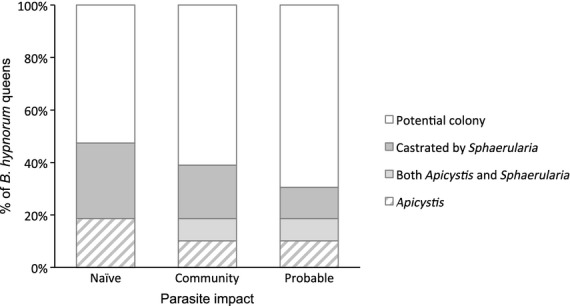
Percentage of *Bombus hypnorum* queens lost from the potential colony-founding population as a result of ‘high-impact’ parasites. *Sphaerularia bombi* and *Apicystis bombi,* are shown additively in ‘naïve’ scenario, with parasite overlap in the ‘community’ scenario and with the actual impact taken into consideration in the ‘probable’ scenario.

The impact of individual parasites on a host population is modified by the structure of the parasite community (Rigaud, Perrot-Minnot & Brown 2010). Consequently, we determined the overall impact of *Apicystis* and *Sphaerularia* on our invasive and native hosts in the context of their parasite community structure. In contrast to an additive scenario, where the impact of parasites might be considered individually, the synergistic scenarios account for co-occurrence of parasite species within hosts. To be conservative, we calculate the community-level impact with and without our knowledge of the differential impact of *Sphaerularia* across species (see above). Under the additive scenario, where the prevalence of high-impact parasites (*Apicystis*,*c*. 19%; *Sphaerularia, c*. 29%) was simply added, *c*. 48% of our *B. hypnorum* queens would be lost from the population of queens potentially able to found a colony (Fig. 4). Under the synergistic ‘community’ scenario, as 8% of *B. hypnorum* queens were infected by both *Sphaerularia* and *Apicystis* (Fig. 3a), *c*. 40% of queens would be lost (11% with only *Apicystis*, 21% with only *Sphaerularia* and 8% with both). As 8% (*N* = 59) of our *B. hypnorum* queens infected with *Sphaerularia* were able to lay eggs they may have been able to produce a colony. Thus, under the synergistic ‘probable’ scenario (Fig. 4) *c*. 32% of our *B. hypnorum* queens would be lost from the population of potential colony-founding queens. For the native species, because *Apicystis* causes early mortality (this study; MacFarlane, Lipa & Liu 1995; Rutrecht & Brown 2008) and *Sphaerularia* causes complete castration (this study; Alford 1969a; MacFarlane, Lipa & Liu 1995; Rutrecht & Brown 2008; Kelly 2009) the community and probable scenarios are identical.

**Figure 5:**
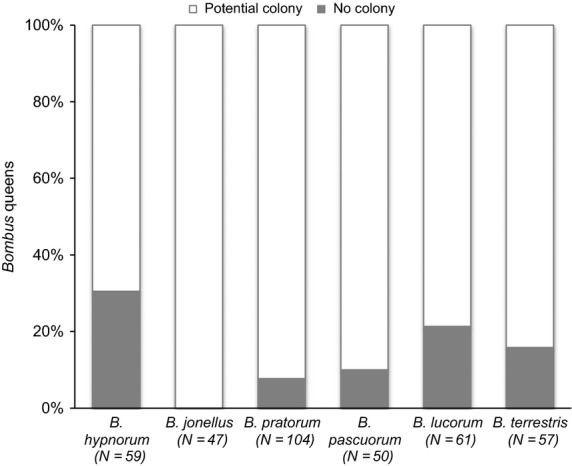
Percentage of *Bombus* queens lost from the potential colony-founding population as a result of the ‘high-impact’ parasites: *Apicystis bombi* and *Sphaerularia bombi*.

If we consider the ‘probable’ impact of *Sphaerularia* and *Apicystis* on the non-native *B. hypnorum* and on the five native bumblebee species (Fig. 5), we find the combined impact of *Apicystis* and/or *Sphaerularia* among bumblebee species differed significantly (Wald = 21·668, d.f. = 5, *P* = 0·001). The combined impact of *Apicycstis* and/or *Sphaerularia* on the non-native *B. hypnorum* was significantly higher than the combined impact of *Apicystis* and/or *Sphaerularia* on *B. jonellus* (Wald = 7·796, d.f. = 1, *P* = 0·005, ExpB = 0·053), *B. pratorum* (Wald = 11·759, d.f. = 1, *P* = 0·001, ExpB = 0·197), *B. pascuorum* (Wald = 8·138, d.f. = 1, *P* = 0·004, ExpB = 0·167) and *B. terrestris* (Wald = 4·636, d.f. = 1, *P* = 0·031, ExpB = 0·346). The combined impact of *Apicycstis* and/or *Sphaerularia* on *B. hypnorum* was not significantly higher than the impact on *B. lucorum* (Wald = 0·946, d.f. = 1, *P* = 0·331, ExpB = 0·658). The combined impact of *Apicycstis* and/or *Sphaerularia* on non-native and native *Bombus* species across sites did not differ significantly (Wald = 9·177, d.f. = 4, *P* = 0·057). Thus, the number of queens lost from the population of queens potentially able to found a colony is higher for *B. hypnorum* (*c*. 32%) than *B. lucorum* (*c*. 23%), *B. terrestris* (*c.* 18%), *B. pascuorum* (*c.* 10%) and *B. pratorum* (*c.* 8%). *Bombus jonellus* were not infected with either *Apicystis* or *Sphaerularia*.

### Functional genetic diversity at the sex-determining locus

Of the 59 *B. hypnorum* queens, 13 produced a colony in the laboratory (i.e. produced one or more live offspring) but of these, three produced 50% male offspring from the first brood indicating that they were producing diploid males (Duchateau, Hoshiba & Velthuis 1994; Gerloff & Schmid-Hempel 2005). In total 59 *B. terrestris* colonies and 57 *B. lucorum* colonies were reared in the laboratory but none of these produced males from the first brood (M. Fürst, pers. comm.). Consequently, the number of sex alleles in the *B. hypnorum* population was estimated to be four, compared to at least 32 for *B. terrestris* and at least 31 for *B. lucorum* (as shown in Table 1) This compares with an estimate of seven sex alleles for 10 Continental European (Scandinavian) *B. hypnorum* colonies (also shown in Table 1) using the same method and data from Brown, Schmid-Hempel & Schmid-Hempel (2003b). Although the estimates for *B. hypnorum* were calculated from a small number of colonies, these estimates indicate that the invading *B. hypnorum* population in the United Kingdom has lower genetic diversity than the native *B. terrestris* and *B. lucorum* populations, and appears to have lower genetic diversity than the Continental European *B. hypnorum* population.

**Table 1:** Estimated number of sex alleles based on the production of diploid males. The transition from significant to non-significant differences gives the minimum number of sex alleles in the population

		Expected		Observed			
	*N*	Diploid	Not Diploid	Diploid	Not Diploid	χ^2^	Significance
*B. terrestris*	31	3·8	55·2	0	59	3·9264	*
*Native*	**32**	**3·7**	**55·3**	**0**	**59**	**3·8198**	**n.s.**
UK							
*B. lucorum*	30	3·8	53·2	0	57	3·931	*
*Native*	**31**	**3·7**	**53·3**	**0**	**57**	**3·8241**	**n.s.**
UK							
*B. hypnorum*	3	8·7	4·3	3	10	4·9983	*
*Invasive*	**4**	**6·5**	**6·5**	**3**	**10**	**2·0319**	**n.s.**
UK							
*B. hypnorum*	6	4·0	6·0	0	10	3·9521	*
*Non-invasive*	**7**	**3·3**	**6·7**	**0**	**10**	**3·3918**	**n.s.**
Scandinavian							

* *P* < 0.05.

‘*N*’ is the number of sex alleles and each row refers to a given number of sex alleles for each species.

The bold shows the minimum estimate for the number of sex alleles in the population.

## Discussion

The successful invasion of the non-native *B. hypnorum* suggested that this species may have escaped from its natural enemies, benefitting from a lower parasite load than native congeners. However, in sharp contrast, we found that not only was *B. hypnorum* infected by the same generalist parasite species as native congeners, but that the prevalence of the high-impact species: *Apicystis* and *Sphaerularia* was also higher (*Apicystis* 19% and *Sphaerularia* 29%) than in native bumblebee species (*Apicystis* 0–7% and *Sphaerularia* 0–20%). These results suggest that enemy release is not the main driver for the successful establishment, range expansion and invasion of this non-native species.

Assessing the impact of parasites on invasive species requires the host to be sufficiently established to provide a large enough sample size for analysis. Despite the fact that our sample area was invaded by this species in 2004, only 3 years after the start of the invasion, this was the first year sufficient spring queens could be caught to enable a comparison of parasite communities between it and native species (M.J.F. Brown, unpublished data). While our samples of *B. hypnorum* were taken from only a portion of its invasive range, given the rapid expansion of this species we believe that our results are likely to be representative of the larger population and provide the first insight into the impact of parasites on invasion in this system.

Before discussing our results further, a number of caveats must be addressed. First, some of our sampled individuals may have been sisters, originating from the same natal colony. This has potential implications for both statistical independence and parasite status. However, given what is known about bumblebee nest density in the United Kingdom (Knight *et al*. 2005) and queen dispersal (Lepais *et al*. 2010), and the low rate at which sisters appear in samples of worker populations (Knight *et al*. 2005), this seems unlikely to be a major concern. Secondly, queens may have emerged from closely aggregated hibernacula, with implications for infection by *Sphaerularia*. While little quantitative data on hibernacula exist (Alford 1969a,b; Sladen 1912, 1989), by sampling florally rich sites (to which spring queens converge) across multiple locations, and collecting sites to exhaustion on sampling visits, our sampling design minimizes this potential bias. Similarly, any unknown impacts of parasites that make queens more or less likely to be caught should have been avoided. Thirdly, by sampling spring queens we were unable to assess escape from social parasites (the cuckoo bumblebees). While the invasive population of *B. hypnorum* has definitely escaped the social parasite *B. norvegicus*, which is absent from the United Kingdom, *B. sylvestris*, another social parasite of *B. hypnorum*, is present. It would be interesting to investigate this host/social–parasite interaction further.

The invading non-native population of *B. hypnorum* supported a similar parasite community at the species level to that of congeneric native host species overall. We note that the potential for non-native parasite strains to be present still exists. Bumblebee parasites can be broadly classified as generalists (MacFarlane, Lipa & Liu 1995; Schmid-Hempel 1998). The most parsimonious explanation, therefore, for this shared community is that *B. hypnorum* acquired its parasites from native hosts. Firstly, given the likely number of foundress queens in the non-native *B. hypnorum* population (based on the number of sex alleles in the population) and the prevalence of parasites in spring queens (MacFarlane, Lipa & Liu 1995; Schmid-Hempel 1998; Rutrecht & Brown 2008) it is highly likely that *B. hypnorum* arrived parasite-free in the United Kingdom, although parasitized queens may have arrived and been unsuccessful in founding a colony. This is supported by the Tasmanian invasion where the low foundress population of *B. terrestris* had a low parasite load (Allen *et al*. 2007). Secondly, the most prevalent parasites in *B. hypnorum* queens were those that either kill queens, or largely prevent colony establishment, thus preventing their potential spread from and within a non-native population (Rutrecht & Brown 2008). The shared parasite community and the hibernation-site transmission route of one of the parasites, *Sphaerularia*, suggest that the invading *B. hypnorum* acquired these parasites in the invaded environment. This matches the predictions of Drake’s model (2003), where release from virulent parasites is important for the establishment phase of the invasion. Interestingly, the parasite community in the non-native *B. hypnorum* was very similar to that of the more abundant congeneric native hosts (*B. pascuorum, B. lucorum* and *B. terrestris*) and much less similar to that of *B. hypnorum*’s closer relatives (*B. jonellus* and *B. pratorum*), suggesting that parasite acquisition was not phylogenetically constrained, but was driven by host abundance. Mechanistically, *B. hypnorum* has probably acquired its parasite community through overlap in the use of floral resources (Durrer & Schmid-Hempel 1994) and hibernation sites (Alford 1969a,b) with the native *Bombus* species. While the number of parasite species infecting *B. hypnorum* was similar to that of native congeners and the parasite community in *B. hypnorum* was similar to the parasite community of the native species overall, prevalence levels, particularly of the high-impact parasites, were higher in the invasive species than in the native species. Higher prevalence could reflect higher susceptibility, which may relate to the low levels of genetic diversity we found in *B. hypnorum* or to maladaptation to the parasites in its new range. Previous studies have shown that inbreeding in bumblebees correlates with higher parasite prevalence (Whitehorn *et al*. 2011), but both mechanisms may be at play. Even though infections by one parasite species, *Sphaerularia*, had a reduced impact in *B. hypnorum*, this was outweighed by its higher prevalence. Nevertheless, the high prevalence and corresponding impact of acquired parasites does not appear to have constrained the spread of *B. hypnorum* across the United Kingdom. However, this high prevalence could still affect the native species. Firstly, higher prevalence in the invasive species may actually reflect a parasite dilution effect, where the presence of the new and possibly more susceptible host has lowered parasite prevalence in native species (Norman *et al*. 1999; Ostfeld & Keesing 2000; Dunn 2009). In the absence of long-term records of parasite prevalence in these, or other bumblebee populations, it is not possible to test this idea. Secondly, the non-native host may also have a detrimental impact on the parasite by preventing transmission. *Sphaerularia* larvae are usually deposited in the soil at hibernation sites by infected queens, where hibernating queens are infected (Lundberg & Svensson 1975), but, if infected queens found colonies, as we found in this study, such deposition at hibernation sites will not occur, and therefore the parasite’s life cycle would be broken, making *B. hypnorum* a dead-end host for *Sphaerularia*. This lack of host competence (Ostfeld & Keesing 2000) is likely to reduce parasite prevalence in native congeners, again through the parasite dilution effect (Ostfeld & Keesing 2000; Dunn 2009). Further studies are needed to determine whether this is in fact happening, and, if so, what quantitative impact it is having on native host–parasite interactions.

In addition to assessing its impact on parasite prevalence, estimating functional genetic diversity at the sex-determining locus enables us to retrospectively assess the number of initial foundress queens in the invasive population (Lundberg & Svensson 1975; Schmid-Hempel *et al*. 2007). *Bombus hypnorum* queens can be polyandrous and mate with between one and six males (Pouvreau 1963; Estoup *et al*. 1995; Schmid-Hempel & Schmid-Hempel 2000; Paxton *et al*. 2001), thus the *B. hypnorum* population in the United Kingdom may have been founded by as few as one or two multiply mated queens. Previous studies of both deliberately introduced populations of bumblebees in New Zealand (Lye, Lepais & Goulson 2011), and introduced *B. terrestris* in Tasmania also found that populations may have been established from as few as one or two mated queens (Schmid-Hempel *et al*. 2007). Although *B. terrestris* (and *B. lucorum*) are usually monandrous, these studies show that bumblebees can establish and become invasive from a small number of founding queens. Finally, diploid-male producing colonies of *B. terrestris* have been shown to have significantly lower fitness under semi-natural conditions (Whitehorn *et al*. 2009), and consequently the high proportion of diploid-male producing *B. hypnorum* colonies found in this study should constrain population expansion.

Nevertheless, despite its high parasite prevalence and low diversity at the sex-determining locus, *B. hypnorum* has rapidly expanded its range in the United Kingdom. What factors might contribute to this success? One contributing factor may be its association with the ‘urban’ environment (urbanization is increasing in Europe, Eigenbrod *et al*. 2011), and its use of resources rarely exploited by other bumblebee species such as nesting sites in trees, bird-boxes and buildings (BWARS; C.M. Jones pers. observ.). *Bombus hypnorum* is also a generalist forager that visits a wide range of flowers (BWARS) and generalists are often associated with biological invasion success (Williamson 1996). Furthermore, *B. hypnorum* has a bivoltine life cycle (producing two generations *per annum*) (Edwards & Jenner 2005) and thus their population might increase more rapidly than univoltine species, such as *B. lucorum* or *B. pascuorum.* In addition, a second generation *B. hypnorum* queen could mate and found a colony without hibernating, thus avoiding possible infection by *Sphaerularia* during hibernation.

A final possible explanation is that the bumblebee species assemblage in Great Britain is depauperate compared with that in Continental Europe, presumably due to the emergence of sea barriers to dispersal at the end of the last Ice Age. In some sense, then, *B. hypnorum* may simply be invading favourable habitat. Similarly, two related *Pyrobombus* species, *B. pratorum* and *B. monticola*, invaded Ireland, in the 1940s and 1970s, respectively, where the bumblebee species assemblage is even more depauperate than Great Britain (Speight 1974; Fitzpatrick *et al*. 2007) suggesting that bees from the *Pyrobombus* sub-genus, such as *B. hypnorum*, may be successful invaders. Unfortunately, no parasite or genetic data exist from the early stages of these invasions to compare with the current study.

Invasion by *B. terrestris* of South America (*c*. 400 km in 8 years, Torretta, Medan & Abrahamovich 2006; Morales *et al*. 2013), an area with a native bumblebee fauna, has proceeded at a similarly rapid rate as *B. hypnorum* in the United Kingdom (*c*. 600 km in 10 years, BWARS). In South America, parasites have been implicated in the invasion success through their impact on the native *Bombus* species (Torretta, Medan & Abrahamovich 2006; Plischuk & Lange 2009; Arbetman *et al*. 2013). Our data from the *B. hypnorum* invasion suggest that it would be extremely valuable to examine the parasite communities and levels of genetic diversity in other invading and native populations to see whether our results are representative of a more general pattern. Unfortunately, while data exist for genetic diversity and parasites in invasive populations in New Zealand and Tasmania (Allen *et al*. 2007; Schmid-Hempel *et al*. 2007; Lye, Lepais & Goulson 2011), the absence of a native bumblebee fauna makes it difficult to extrapolate these results to other areas.

To conclude, this study shows that high parasite impact and low functional genetic diversity at the sex-determining locus have not prevented the invasion of a non-native bumblebee. This not only has implications for understanding economically important and ecologically devastating invasions (Inoue, Yokoyama & Washitani 2008; Plischuk & Lange 2009; Arbetman *et al*. 2013), it also has implications for the successful design of re-introduction programs which begin with low founding populations and low parasite load (IUCN; Frankham, Ballou & Briscoe 2010). While the obvious next steps would be to investigate *B. hypnorum* in its native range, or the parasite community and genetic diversity of other invasive *Bombus* species in their invaded ranges, this work provides an important step in understanding the role of parasites and genetic variation in insect invasions. A recent study (Venesky *et al*. 2012) suggested that captive breeding programs for re-introductions should select for tolerance to natural enemies, to avoid the impact of such enemies in small re-introduced populations with low genetic diversity. Our results, where a genetically depauperate, invasive population has expanded despite high parasite impact, suggest that such complex selection may not be required.
